# Thrombotic and Hypercoagulability Complications of COVID-19: An Update

**DOI:** 10.2147/JBM.S316014

**Published:** 2021-08-31

**Authors:** Sapha Shibeeb, Muneera Naseer Ahmad

**Affiliations:** 1 Department of Biomedical Science, College of Health Sciences, QU Health, Qatar University, Doha, Qatar; 2 Biomedical and Pharmaceutical Research Unit, QU Health, Qatar University, Doha, Qatar

**Keywords:** COVID-19, SARS-CoV-2, thrombosis, thromboembolism

## Abstract

The current COVID-19 pandemic emerged in December 2019, in China, affecting millions of people worldwide. COVID-19 is mainly a disease of the respiratory system, yet systematic complications have also been reported among SARS-CoV-2 infected patients. Thrombotic complications are one of the severe clinical outcomes of COVID-19, especially among critically ill patients, and are associated with poor prognosis. To date, many studies have concluded that COVID-19 increases the incidence of thrombotic events and coagulopathies; however, the exact mechanism behind such a disease outcome is not well known. Various pathophysiological mechanisms for thrombotic events in COVID-19 have been proposed, these include virus-induced endothelial cell damage, inflammation, and excess production of pro-inflammatory cytokines. As a result, most critically diseased COVID-19 patients are managed with prophylactic anticoagulant, yet some still develop thrombotic episodes. Therefore, better understanding of the mechanisms behind the thrombotic complications is needed to develop treatments that specifically target such pathways, which may aid in better disease management and improve the prognosis.

## Introduction

In December 2019, the Chinese Center for Disease Control and Prevention identified the presence of a novel coronavirus in throat swab samples from a series of pneumonia cases of unknown etiology, that presented with dry cough, dyspnea, and fever.^1^ The newly-identified virus was named as Severe Acute Respiratory Syndrome Coronavirus 2 (SARS-CoV-2), since it resembled SARS-CoV that emerged in 2002–2003 and resulted in high morbidities and mortalities. By March 2020, the World Health Organization declared a global pandemic, and the disease caused by the virus was named Coronavirus disease 2019 (COVID-19).^2^ Subsequently, many countries around the world implemented various preventive strategies to minimize the rate of the virus spread. Yet, SARS-CoV-2 affected millions of different age groups worldwide. The latest statistics show the infected cases exceeded 158 million with more than 3 million deaths worldwide.

**Figure 1:**
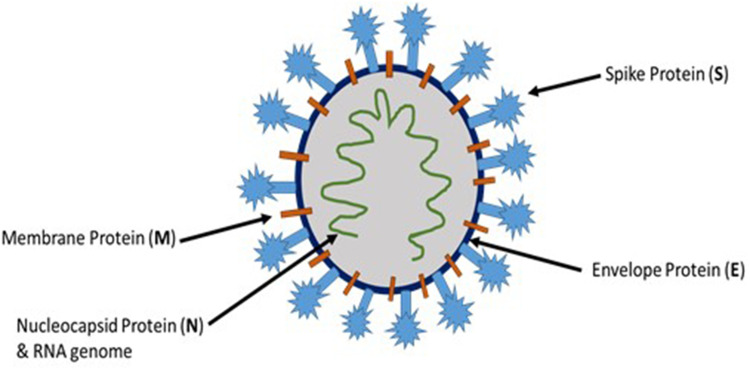
Schematic representation of SARS-CoV-2 structure.

The novel coronavirus, SARS-CoV-2, belongs to the coronaviridae family, which are positive-sense single-stranded enveloped RNA viruses. There are four different genera for coronaviruses, and SARS-CoV-2 belongs to the β coronaviruses.^3^ SARS-CoV-2 and coronaviruses in general have four essential structural proteins (Figure 1), namely spike (S) glycoprotein, envelope (E) glycoprotein, membrane (M) glycoprotein, and nucleocapsid (N) protein. These proteins are necessary for successful viral attachment and penetration of the host cells, synthesis of viral proteins, maturation, and release of the viral progeny.^4^ The viral S protein, a transmembrane protein that protrudes from the viral surface, has been considered as an important factor in the pathogenesis of coronaviruses including SARS-CoV-2. S glycoprotein aids in the viral attachment and fusion to the host cell membrane.^5^

Thrombotic episodes are one of the severe complications of COVID-19, particularly in critically ill patients, which can be associated with poor prognosis. A growing body of evidence suggests that COVID-19 increases the incidence of thrombotic complications; however, the exact mechanisms are not fully elucidated. Therefore, the current review aims to provide an overview of the available literature to outline the current knowledge of the risks, pathogenesis, and the therapeutic interventions for thrombotic complications that are associated with the COVID-19 pandemic.

### Pathogenesis of COVID-19 Infection

SARS-CoV-2 is a zoonotic virus that evolved in a way to be able to be transmitted from human-to-human.^6^ SARS-CoV-2 can transmit from one individual to another either by direct transfer through aerosols or indirectly via contaminated surfaces. SARS-CoV-2 contains large RNA genomes flanked by 5′ and 3′ untranslated regions containing cis-acting secondary RNA structures necessary for RNA synthesis and replication. At the 5′ end, the viral RNA possesses two large open reading frames (ORFs; ORF1a and ORF1b) that occupy two-thirds of the nonstructural genome.^7^ The currently proposed pathogenesis of SARS-CoV-2 is derived from the knowledge that is available on SARS-CoV, where the viral spike protein binds to host’s angiotensin-converting enzyme 2 (ACE2) receptor, which is differentially expressed on various tissues, including respiratory tract cells, gastrointestinal tract cells, cardiac muscle cells, and endothelial cells.^8^

**Figure 2:**
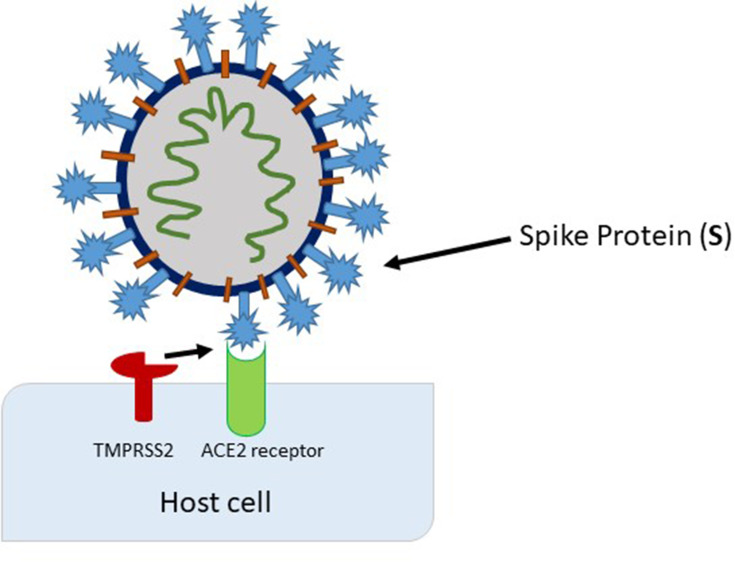
Activation of the spike protein by TMPRSS2 at (or close to) the cell surface, leading to fusion of the viral membrane with the plasma membrane.

A successful binding of the spike protein to ACE2 and the internalization of the virus requires the presence of the specific protease transmembrane serine protease 2 (TMPRSS2) that cleaves and activates ACE2.^9^ With the successful viral entry into the cells, host replication machinery is used, and viral progenies are produced, which can further infect adjacent cells (Figure 2). Indeed, recent evidence shows TMPRSS2 is expressed in human endothelial cells obtained from the lungs which may explain the pulmonary complications of COVID-19.^10^ Furthermore, smokers and patients with moderate-to-severe COPD had been found to have higher levels of ACE2 mRNA and protein levels in lung tissue. Furthermore, hypertensive patients were found to have the more severe form of the disease, suggesting antihypertensive therapy may play a role in developing serious COVID-19 infection due to enhanced ACE2 and SARS-CoV-2 interactions.^11^

Upon entry, the viral genome is released into the cytosol, this leads to the immediate translation of ORF1a and ORF1b which encode for 15–16 non-structural proteins. The majority of these non-structural proteins make up the viral replication and transcription complex (TRC). In turn, the TRC houses RNA-processing and modifying enzymes as well as a RNA proofreading mechanism that is essential for maintaining the integrity of the viral genome. Translation of ORF1a and ORF1b results in polyproteins that are co-translationally and post-translationally processed into the single non-structural proteins, which leads to the formation of the viral replication and transcription system. As non-structural proteins are formed and expressed, the synthesis of viral replication machinery also takes place, which consists of perinuclear double-membrane vesicles (DMVs), convoluted membranes (CMs), and small open double-membrane spherules (DMSs) forming a protective and suitable microenvironment for viral genomic RNA replication and transcription of subgenomic mRNAs. The newly synthesized viral genomic RNA results in budding into the lumen of secretory vesicular compartments, which are then secreted from the infected cell by exocytosis.^7^,^12^

The clinical presentations of COVID-19 are highly variable from one individual to another. COVID-19 ranges from asymptomatic to severe disease that could result in death. However, mild-to-moderate flu-like symptoms are the most common presentations among COVID-19 patients, including fever, dry cough, sore throat, runny nose, and, in some cases, involvement of the lower respiratory tract that may lead to acute respiratory distress syndrome (ARDS).^13^ Other general symptoms such as weakness, headache, and gastrointestinal symptoms including diarrhea and vomiting have also been reported.^14^ Moreover, to a lower extent some of the COVID-19 patients experienced loss of smell and taste.^15^ Some affected individuals could further progress to severe forms of the disease, where they develop severe pneumonia, pulmonary edema, septic shock, and organ failure that would result in death.^16^ Thromboembolic events and acute kidney injury have also been reported as COVID-19 complications.^13^ Indeed, laboratory findings such as thrombocytopenia, increased prothrombin time, and activated partial thromboplastin time along with elevated D-dimer were reported among critically ill patients, which further supports the thrombotic and coagulopathy complications in COVID-19 patients. Together these findings indicate that COVID-19 is a systematic disease with serious thrombotic complications that require more attention.

### Thrombotic Complications of SARS-CoV-2 Infection

A number of studies have reported thrombotic and hypercoagulability complications among COVID-19 cases. These complications represent a serious worsening of the disease since it is associated with adverse outcomes. A recent systematic review reported that among 2,928 COVID-19 severely diseased patients, 56.3% developed thrombosis, and 34% of ICU admitted patients were found to have thrombotic complications, where 16.1% were reported with deep vein thrombosis and 12.6% with pulmonary embolism.^17^ Many other independent studies have reported different rates of thrombotic complications among COVID-19 patients (Table 1). It is now established that critically ill COVID-19 patients were associated with a higher incidence of thrombotic events than other COVID-19 patients. This could be associated with higher levels of pro-inflammatory and dysregulated fibrinolytic states among severe COVID-19 cases.

**Table 1:** Incidence of Thrombotic Complications Among COVID-19 Patients

Author	Number of Subjects, N	Incidence of Thrombotic Event	Type of Thrombotic Event
Jenner et al^17^	2,928	90.3% experienced thrombotic complications (34% of ICU patients 56.3% non-ICU patients)	Deep vein thrombosis (DVT) reported (16.1% of ICU patients) Pulmonary embolism (12.6% of ICU patients) Venous thrombosis (56.3% routine screening)
Chen et al^56^	88	46%	Deep vein thrombosis
Shah et al^57^	187	N=81 (43.3%)	Pulmonary embolism (22.5%) Deep vein thrombosis (11.8%) Arterial complications (13.4%)
Gibson et al^58^	72	N=12 (16.7%)	Lower extremity of deep vein thrombosis
Monfardini et al^59^	34	N=26 (76%)	Pulmonary thromboembolism
Avruscio et al^60^	85 (41 critical cases)	N=43 (50.6%)	Pulmonary embolism (9.8%) Deep vein thrombosis (42.4%) Superficial vein thrombosis (3.5%)
Al-Samkari et al^61^	400 (144 critically ill)	N=38	Venous thromboembolism Arterial thrombosis
Demelo-Rodriguez et al^62^	156	N=23	Deep vein thrombosis
Piazza et al^63^	1,114	N=66	Venous thromboembolic event (4.6%) Deep vein thrombosis (3.5%) Pulmonary embolism (0.7%) Disseminated intravascular coagulation (1.3%)

Indeed, it was reported in a cohort of 54 COVID-19 patients who died from the infection, it was found those patients had higher levels of D-dimer, troponin, and interleukin-6 (IL-6).^18^ Furthermore, Wang et al^19^ reported that, among 199 COVID-19 patients, elevated levels of D-dimer, fibrinogen degradation products, prolonged prothrombin time, and thrombin time were found to be higher among patients with severe disease manifestations. These findings indicate that COVID-19 is associated with abnormal coagulation and subsequently leads to thrombotic complications and severe disease outcome. Moreover, Harenberg et al^20^ reported there was an initial increase in the prothrombin time and activated partial thromboplastin time, but later both are decreased due to consumption of coagulation factors. These findings were also associated with an increase in the platelet count, fibrinogen, and D-dimer. In addition, in a cohort of 248 COVID-19 cases, Yao et al^21^ reported that D-dimer levels were higher among 185 patients, and it was significantly increasing with an increase in disease severity.

Interestingly, the ABO blood group system has also been suggested to contribute to susceptibility and severity of COVID-19 infection. Indeed, a study by Marcos et al^22^ concluded that blood group O was associated with lower ICU admission and thrombotic complications when compared to other blood groups. Furthermore, blood group B had significantly higher rates of thrombotic complications and was associated with more ICU. A possible mechanism for this is that plasma levels of Von Willebrand factor ((VWF) a glycoprotein essential for thrombus formation) levels are lower in group O patients when compared to non-O blood group patients.

### Pathogenesis of Thrombotic Complications of COVID-19

Various factors have been reported to be associated with increased incidence of coagulopathies among COVID-19 patients. These include patients’ age, and coexistence of chronic disease such as cardiovascular disease, diabetes, and hypertension.^23^ Indeed, COVID-19 patents with chronic disease such as diabetes have been shown to have increased pro-inflammatory mediators IL-6, suggesting these patients are at higher risk of developing more serious complications of COVID-19.^24^,^25^ In addition, males have been shown to have been more at risk of developing thrombotic complications. Indeed, various COVID-19 reports have so far shown that elderly patients are severely affected by the infection and they account for most of the severe cases that requires ICU admission, with a higher incidence of thrombosis.^26^

The exact mechanism responsible for COVID-19 induced thrombosis is not fully understood. However, several mechanisms have been put forward in an attempt to explain these complications among COVID-19 patients. Such mechanisms include increased platelet activation and activation coagulation cascade and/or decreased fibrinolysis, in addition to immune activation. However, a central mechanism involves virus-induced endothelial damage. Endothelial cells express the ACE2 receptor which is a SARS-CoV-2 target receptor. Ut has been suggested that viral penetration of endothelial cells inflicts tissue damage and subsequently exposure of collagen, a potent platelet agonist, leading to platelet adhesion and activation, furthermore, injured endothelial cells release tissue factor.^27^,^28^ Initially this leads to the activation and the recruitment of platelets, and further release of platelet granules content that aid in the platelet plug formation,^29^ whilst tissue factor activates the extrinsic pathway of the coagulation cascade. Subsequently this leads to the activation of the intrinsic pathway of coagulation cascade, formation of thrombin that converts fibrinogen to fibrin which further stabilizes platelet plug, and forms thrombus.^28^,^30^ Evidence of increased ACE2 expression on the endothelial cells of SARS-CoV-2 infected patients has been reported, which suggest a higher probability of endothelial cells damage that leads to thrombotic outcome.^31^

Abnormal fibrinolysis has also been suggested to contribute to thrombotic events during COVID-19 infection. Wright et al^32^ reported that among 44 ICU admitted patients, 57% experienced a complete lack in the lysis of the clots, suggesting defective fibrinolysis. This was later supported by the increased levels of plasminogen activator inhibitor 1 among COVID-19 patients, which inhibits the plasminogen activator and leads to a decrease in the fibrin degradation.^33^

**Figure 3:**
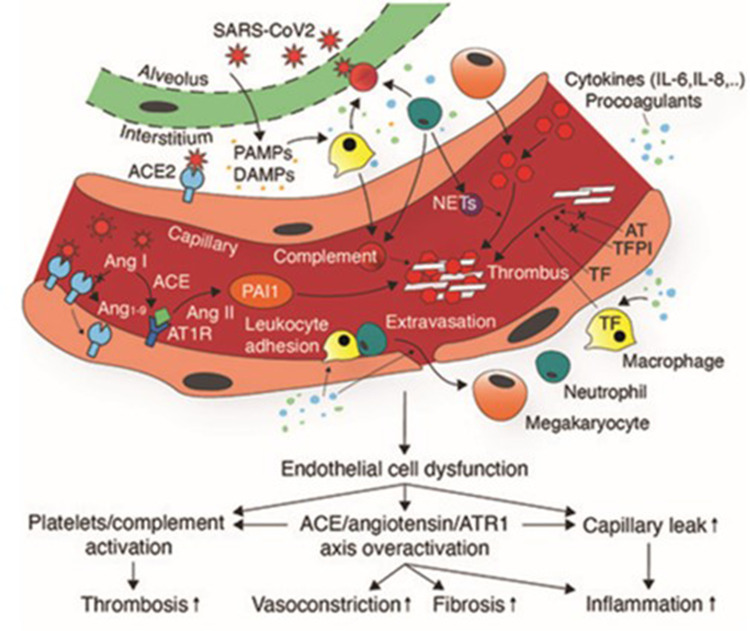
Pathogenesis of thrombotic complications of COVID-19. SARS-CoV2 infection induces endothelial damage triggering endothelial release of cytokines, increasing capillary permeability. PAMPs and DAMPs induced activation of neutrophils, and macrophages results in localized production of cytokines, procoagulants, and complement activation, leading to further endothelial damage and tissue factor release. Endothelial damage exposes collagen and other prothrombotic mediators leading to thrombus formation.

**Notes:** Adapted from Brosnahan SB, Jonkman AH, Kugler MC, Munger JS, Kaufman DA. COVID-19 and Respiratory System Disorders: Current Knowledge, Future Clinical and Translational Research Questions. Arterioscler Thromb Vasc Biol. 2020;40(11):2586–2597.^64^ © 2020 The Authors. Creative Commons Attribution License (https://creativecommons.org/licenses/by/4.0/legalcode). https://www.ahajournals.org/doi/pdf/10.1161/ATVBAHA.120.314515?download=true.

The immune system and homeostasis complement each other in order to defend against a pathogen and prevent further dissemination of the pathogen, by a process known as immuno-thrombosis. This physiological mechanism could be dysregulated, leading to the formation of excess thrombus formation.^34^ When endothelial cells are infected with SARS-CoV-2, they undergo pyroptosis, a form of cell death.^35^ This process is associated with the release of cellular content such as pathogen associated molecular pattern (PAMPs), and damage associated molecular patterns (DAMPs) causing inflammation. DAMPs and PAMPs interact with pattern recognition receptor and Toll-like receptor on innate immune cells triggering immune response and the release of pro-inflammatory cytokines and this enhances the expression of tissue factor.^35^,^36^ Therefore, some patients develop an induced immune response to SARS-CoV-2 that is defined as cytokine storm.^37^ COVID-19 patients are found to have increased levels of specific chemokines and cytokines, which include IL-6, interferon-gamma, and IL-2.^38^ IL-6 is reported to increase the production of platelets in COVID-19 patients, enhances the expression of tissue factor on monocytes and endothelial cells, and by itself can lead to further endothelial damage.^38^ To a similar extent it has been reported that interferon-gamma also increases the production of platelets and causes endothelial dysfunction, creating a prothrombotic status. On the other hand, IL-2 in COVID-19 patients is reported to increase the production of plasminogen activator inhibitor-1, which reduces fibrinolysis^39^ (Figure 3).

In addition to these proposed mechanisms, the complement system has also been implicated in the development of thrombotic complications in COVID-19 patients. Indeed, in a study conducted by Magro et al,^40^ it was found that there is a continuous activation of the alternative and the lectin pathways of the complement system, that was supported by the presence of terminal C5b-9 complement complex on the vessels of the affected individual’s lungs. The membrane attack complex, C5b-9, has the ability to activate platelets, endothelial damage, and enhance the release of VWF. Individual complement components, including C5a, have the ability to induce the production of tissue factor, plasminogen activator inhibitor-1 and IL-6, which further stimulate thrombus formation.^41^ Furthermore, since SARS-CoV-2 invades the cells through an ACE2 receptor, this causes downregulation of the receptor and subsequent upregulation of angiotensin II, which is normally degraded by ACE2. High levels of angiotensin II cause vasocontraction,^42^ increased levels of tissue factor, and plasminogen activator inhibitor, therefore favoring a hypercoagulable state.^43^

### Clinical Manifestation of Thrombotic Events in COVID-19 Patients

Thrombotic complications of COVID-19 have been reported from the start of this pandemic and are highly reported among severely ill patients.^17^ Severe infection with SARS-CoV-2 has been reported to be associated with the development of microvascular and macrovascular thrombosis that eventually leads to an increase in the mortality rates. Macrovascular and microvascular thrombosis results from platelet activation and the activation of the coagulation cascade and subsequent thrombus formation in blood vessels of varying sizes that could cause partial or complete occlusion of the blood vessels.^44^,^45^ Venous thromboembolism and arterial thrombosis are among the macrovascular thrombotic complications of COVID-19. Both forms of venous thromboembolism, pulmonary embolism, and deep vein thrombosis have been reported among COVID-19 patients. It was reported that among 1,765 patients the rate of venous pulmonary embolism in COVID-19 patients was around 22%, with higher incidence rates among ICU admitted patients.^46^ Whilst some patients experienced deep vein thrombosis. Zhang et al^47^ reported that among 143 hospitalized COVID-19 patients, 66 (46.2%) developed deep vein thrombosis, and it was common in elderly patients and was associated with poor disease outcome and higher mortality rates. These rates could be even higher than the reported data, since not all COVID-19 patients undergo computerized tomography, mainly because of the risk of spreading the infection. Moreover, a meta-analysis involved 46,348 COVID-19 cases reported that around 7% had increased levels of troponin which is associated with myocardial injury is also reported among COVID-19 patients.^48^ Identifying the risks and incidence rate of these complications among COVID-19 is essential for determining the best procedures for diagnosing such outcomes for best patient’s management. Furthermore, COVID-19 patients also experience coagulation abnormalities that are similar but not identical to thrombotic thrombocytopenic purpura, or hemolytic uremic syndrome or disseminated intravascular coagulation (DIC). Higher levels of D-dimer, thrombocytopenia and prolonged prothrombin time in COVID-19 patients suggest the occurrence of DIC, yet in DIC the levels of D-dimer is higher than that of COVID-19 and thrombocytopenia is more predominant. In addition, COVID-19 patients with thrombotic complications have high levels of ferritin and lactate dehydrogenase along with platelet rich plaques in the lungs and other organs, yet there is no schistocytes that are normally present in the case of thrombotic microangiopathies.^49^ This indicates that thrombotic manifestation in COVID-19 have unique features and the mechanism behind this variation need to be investigated for better disease diagnosis and management.

### Management of COVID-19 Associated Thrombotic Complications

Due to the rapidly evolving literature, there are no unified guidelines on how best to diagnose and manage thrombotic and hypercoagulability in COVID-19 patients. This is partly due to the variability in incidence among studies which can be attributed to differences in population. However, most of the published guidelines recommend using prophylactic anticoagulant for all hospitalized COVID-19 patients, to avoid such severe disease outcomes.^50^ Most of the guidelines suggest the use of daily low-molecular-weight heparins or subcutaneous unfractionated heparin. Low-molecular-weight heparin is considered more beneficial than other prophylactic regimens, particularly in COVID-19 cases, as it has been previously reported that it has a longer half-life than unfractionated heparin,^51^ acts as an inhibitor for viral attachment by binding to SARS-CoV-2 spike protein,^52^ and it has immunomodulatory and anti-inflammatory effects.^53^ Despite using thromboprophylaxis, some critically ill patients still developed thrombotic complications. This could be due to heparin resistance and lower levels of anti-activated factor X in these patients, which could be attributed to high levels of fibrinogen and reduced antithrombin levels in COVID-19 patients, yet the exact mechanism for heparin resistance among COVID-19 patients is not known.^54^ Therefore, it is recommended to use higher doses of prophylactic anticoagulant, as it was reported that with higher doses the levels of anti-activated factor X are higher, which could aid in preventing thrombosis. Yet, this finding cannot be generalized since it is not known if all COVID-19 hospitalized subjects would benefit from higher doses of thromboprophylaxis without leading to additional complications.

Venous thromboembolism is mainly managed by the use of therapeutic anticoagulants. The best choice of the anticoagulant as a treatment depends on patients’ renal and hepatic functions, gastrointestinal function, and thrombocytopenia. Furthermore, for patients admitted to hospital and receiving direct care it is preferred to use low-molecular weight heparin, unfractionated heparin (UFH), or fondaparinux (particularly for patients with heparin-induced thrombocytopenia), but for out-patients it is better to use direct oral anticoagulants. In addition, a number of drugs have been studied to be used as a treatment for sepsis-induced thrombotic complications, and it is suggested that they might have a therapeutic role in COVID-19 and these include; Danaparoid, that weakens thrombin production, Sulodexide, which potentiates anti-proteolytic activity of anti-thrombin and heparin co-factor, antithrombin, that inactivates coagulation enzymes, and thrombomodulin, which acts as a co-factor for thrombin.^55^

## Conclusion

SARS-CoV-2 infection rates are still increasing with an increase in the morbidity and mortality rates. There is accumulating evidence that COVID-19 is associated with various systematic complications, including thrombotic and hypercoagulability. Yet, the exact mechanism of such complication is not fully understood. However, a central mechanism that has been postulated is SARS-CoV-2 induced endothelial damage via ACE2 receptor. Endothelial injury and inflammation result in a series of reactions leading to the activation of the coagulation pathways and platelet. Moreover, increased levels of pro-inflammatory mediators complement cascade activation and shut down of anti-thrombotic pathways have been shown to play a role in the pathogenesis of thrombotic complications. However, the available data are limited, and further investigations are required for better understanding of the disease mechanisms.

**Acknowledgment**

Open access funding was provided by Qatar National Library.

## References

[1] Lu H, Stratton CW, Tang Y-W. Outbreak of pneumonia of unknown etiology in Wuhan, China: the mystery and the miracle. *J Med Virol*. 2020;92(4):401–402. doi:10.1002/jmv.25678 31950516 PMC7166628

[2] Sohrabi C, Alsafi Z, O’Neill N, et al. World health organization declares global emergency: a review of the 2019 novel coronavirus (COVID-19). *Int J Surg*. 2020;76:71–76. doi:10.1016/j.ijsu.2020.02.034 32112977 PMC7105032

[3] Phan T. Novel coronavirus: from discovery to clinical diagnostics. *Infect Genet Evol*. 2020;79:104211. doi:10.1016/j.meegid.2020.104211 32007627 PMC7129799

[4] Satarker S, Nampoothiri M. Structural proteins in severe acute respiratory syndrome coronavirus-2. *Arch Med Res*. 2020;51(6):482–491. doi:10.1016/j.arcmed.2020.05.012 32493627 PMC7247499

[5] Fehr AR, Perlman S. Coronaviruses: an overview of their replication and pathogenesis. *Methods Mol Biol*. 2015;1282:1–23. doi:10.1007/978-1-4939-2438-7_1 25720466 PMC4369385

[6] Wong ACP, Li X, Lau SKP, Woo PCY. Global epidemiology of bat coronaviruses. *Viruses*. 2019;11(2):174. doi:10.3390/v11020174 PMC6409556 30791586

[7] V’kovski P, Kratzel A, Steiner S, Stalder H, Thiel V. Coronavirus biology and replication: implications for SARS-CoV-2. *Nat Rev Microbiol*. 2021;19(3):155–170. doi:10.1038/s41579-020-00468-6 33116300 PMC7592455

[8] Zou X, Chen K, Zou J, Han P, Hao J, Han Z. Single-cell RNA-seq data analysis on the receptor ACE2 expression reveals the potential risk of different human organs vulnerable to 2019-nCoV infection. *Front Med*. 2020;14(2):185–192. doi:10.1007/s11684-020-0754-0 32170560 PMC7088738

[9] Hoffmann M, Kleine-Weber H, Schroeder S, et al. SARS-CoV-2 cell entry depends on ACE2 and TMPRSS2 and is blocked by a clinically proven protease inhibitor. *Cell*. 2020;181(2):271–80.e8. doi:10.1016/j.cell.2020.02.052 32142651 PMC7102627

[10] Matarese A, Gambardella J, Sardu C, Santulli G. miR-98 regulates TMPRSS2 expression in human endothelial cells: key implications for COVID-19. *Biomedicines*. 2020;8(11):462. doi:10.3390/biomedicines8110462 PMC7693865 33143053

[11] Sardu C, Maggi P, Messina V, et al. Could anti‐hypertensive drug therapy affect the clinical prognosis of hypertensive patients with COVID‐19 infection? data from centers of southern Italy. *J Am Heart Assoc*. 2020;9(17):e016948. doi:10.1161/JAHA.120.016948 32633594 PMC7660768

[12] Sanyal S. How SARS-CoV-2 (COVID-19) spreads within infected hosts—what we know so far. *Emerging Topics Life Sci*. 2020;4(4):383–390. doi:10.1042/ETLS20200165 PMC7733667 33269805

[13] Huang C, Wang Y, Li X, et al. Clinical features of patients infected with 2019 novel coronavirus in Wuhan, China. *Lancet*. 2020;395(10223):497–506. doi:10.1016/s0140-6736(20)30183-5 31986264 PMC7159299

[14] Shi H, Han X, Jiang N, et al. Radiological findings from 81 patients with COVID-19 pneumonia in Wuhan, China: a descriptive study. *Lancet Infect Dis*. 2020;20(4):425–434. doi:10.1016/S1473-3099(20)30086-4 32105637 PMC7159053

[15] Agyeman AA, Chin KL, Landersdorfer CB, Liew D, Ofori-Asenso R. Smell and taste dysfunction in patients with COVID-19: a systematic review and meta-analysis. *Mayo Clin Proc*. 2020;95(8):1621–1631. doi:10.1016/j.mayocp.2020.05.030 32753137 PMC7275152

[16] Tabata S, Imai K, Kawano S, et al. Clinical characteristics of COVID-19 in 104 people with SARS-CoV-2 infection on the diamond princess cruise ship: a retrospective analysis. *Lancet Infect Dis*. 2020;20(9):1043–1050. doi:10.1016/S1473-3099(20)30482-5 32539988 PMC7292609

[17] Jenner WJ, Kanji R, Mirsadraee S, et al. Thrombotic complications in 2928 patients with COVID-19 treated in intensive care: a systematic review. *J Thromb Thrombolysis*. 2021;51(3):595–607. doi:10.1007/s11239-021-02394-7 33586113 PMC7882250

[18] Zhou F, Yu T, Du R, et al. Clinical course and risk factors for mortality of adult inpatients with COVID-19 in Wuhan, China: a retrospective cohort study. *Lancet (London, England)*. 2020;395(10229):1054–1062. doi:10.1016/S0140-6736(20)30566-3 PMC7270627 32171076

[19] Wang J, Zhang H, Qiao R, et al. Thrombo-inflammatory features predicting mortality in patients with COVID-19: the FAD-85 score. *J Int Med Res*. 2020;48(9):300060520955037. doi:10.1177/0300060520955037 32960106 PMC7511832

[20] Harenberg J, Favaloro E. COVID-19: progression of disease and intravascular coagulation - present status and future perspectives. *Clin Chem Lab Med*. 2020;58(7):1029–1036. doi:10.1515/cclm-2020-0502 32406381

[21] Yao Y, Cao J, Wang Q, et al. D-dimer as a biomarker for disease severity and mortality in COVID-19 patients: a case control study. *J Intensive Care*. 2020;8(1):49. doi:10.1186/s40560-020-00466-z 32665858 PMC7348129

[22] Marcos SZ, Antelo ML, Galbete A, Etayo M, Ongay E, García-Erce JA. Infection and thrombosis associated with COVID-19: possible role of the ABO blood group. *Medicina Clínica (English Edition)*. 2020;155(8):340–343. doi:10.1016/j.medcle.2020.06.013 33015369 PMC7519708

[23] Fang L, Karakiulakis G, Roth M. Are patients with hypertension and diabetes mellitus at increased risk for COVID-19 infection? *Lancet Respir Med*. 2020;8(4):e21. doi:10.1016/S2213-2600(20)30116-8 32171062 PMC7118626

[24] Sardu C, Gargiulo G, Esposito G, Paolisso G, Marfella R. Impact of diabetes mellitus on clinical outcomes in patients affected by Covid-19. *Cardiovasc Diabetol*. 2020;19(1):1–4. doi:10.1186/s12933-020-01047-y 32527257 PMC7289072

[25] Sardu C, D’Onofrio N, Balestrieri ML, et al. Hyperglycaemia on admission to hospital and COVID-19. *Diabetologia*. 2020;63(11):2486–2487. doi:10.1007/s00125-020-05216-2 32632527 PMC7335920

[26] Liu K, Chen Y, Lin R, Han K. Clinical features of COVID-19 in elderly patients: a comparison with young and middle-aged patients. *J Infect*. 2020;80(6):e14–e18. doi:10.1016/j.jinf.2020.03.005 PMC7102640 32171866

[27] Ackermann M, Verleden SE, Kuehnel M, et al. Pulmonary vascular endothelialitis, thrombosis, and angiogenesis in covid-19. *N Engl J Med*. 2020;383(2):120–128. doi:10.1056/NEJMoa2015432 32437596 PMC7412750

[28] Gale AJ. Continuing education course# 2: current understanding of hemostasis. *Toxicol Pathol*. 2011;39(1):273–280. doi:10.1177/0192623310389474 21119054 PMC3126677

[29] Golebiewska EM, Poole AW. Platelet secretion: from haemostasis to wound healing and beyond. *Blood Rev*. 2015;29(3):153–162. doi:10.1016/j.blre.2014.10.003 25468720 PMC4452143

[30] Bautista-Vargas M, Bonilla-Abadía F, Cañas CA. Potential role for tissue factor in the pathogenesis of hypercoagulability associated with in COVID-19. *J Thromb Thrombolysis*. 2020;50:479–483. doi:10.1007/s11239-020-02172-x 32519164 PMC7282470

[31] Yuki K, Fujiogi M, Koutsogiannaki S. COVID-19 pathophysiology: a review. *Clin Immunol*. 2020;215:108427. doi:10.1016/j.clim.2020.108427 32325252 PMC7169933

[32] Wright FL, Vogler TO, Moore EE, et al. Fibrinolysis shutdown correlation with thromboembolic events in severe COVID-19 infection. *J Am Coll Surg*. 2020;231(2):193–203. doi:10.1016/j.jamcollsurg.2020.05.007 32422349 PMC7227511

[33] Goshua G, Pine AB, Meizlish ML, et al. Endotheliopathy in COVID-19-associated coagulopathy: evidence from a single-centre, cross-sectional study. *Lancet Haematol*. 2020;7(8):e575–e82. doi:10.1016/S2352-3026(20)30216-7 32619411 PMC7326446

[34] Engelmann B, Massberg S. Thrombosis as an intravascular effector of innate immunity. *Nat Rev Immunol*. 2013;13(1):34–45. doi:10.1038/nri3345 23222502

[35] Tay MZ, Poh CM, Rénia L, MacAry PA, Ng LFP. The trinity of COVID-19: immunity, inflammation and intervention. *Nat Rev Immunol*. 2020;20(6):363–374. doi:10.1038/s41577-020-0311-8 32346093 PMC7187672

[36] Puelles VG, Lütgehetmann M, Lindenmeyer MT, et al. Multiorgan and renal tropism of SARS-CoV-2. *New Engl J Med*. 2020;383(6):590–592. doi:10.1056/NEJMc2011400 32402155 PMC7240771

[37] Liu Y, Yang Y, Zhang C, et al. Clinical and biochemical indexes from 2019-nCoV infected patients linked to viral loads and lung injury. *Sci China Life Sci*. 2020;63(3):364–374. doi:10.1007/s11427-020-1643-8 32048163 PMC7088566

[38] Wang J, Jiang M, Chen X, Montaner LJ. Cytokine storm and leukocyte changes in mild versus severe SARS‐CoV‐2 infection: review of 3939 COVID‐19 patients in China and emerging pathogenesis and therapy concepts. *J Leukoc Biol*. 2020;108(1):17–41. doi:10.1002/JLB.3COVR0520-272R 32534467 PMC7323250

[39] Du F, Liu B, Zhang S. COVID-19: the role of excessive cytokine release and potential ACE2 down-regulation in promoting hypercoagulable state associated with severe illness. *J Thromb Thrombolysis*. 2020;51:1–17. 10.1007/s11239-020-02224-2 PMC7365308 32676883

[40] Magro C, Mulvey JJ, Berlin D, et al. Complement associated microvascular injury and thrombosis in the pathogenesis of severe COVID-19 infection: a report of five cases. *Transl Res*. 2020;220:1–13. doi:10.1016/j.trsl.2020.04.007 32299776 PMC7158248

[41] Fletcher-Sandersjöö A, Bellander B-M. Is COVID-19 associated thrombosis caused by overactivation of the complement cascade? A literature review. *Thromb Res*. 2020;194:36–41. doi:10.1016/j.thromres.2020.06.027 32569879 PMC7301826

[42] Forrester SJ, Booz GW, Sigmund CD, et al. Angiotensin II signal transduction: an update on mechanisms of physiology and pathophysiology. *Physiol Rev*. 2018;98(3):1627–1738. 29873596 10.1152/physrev.00038.2017 PMC6335102

[43] Celi A, Cianchetti S, Dell’Omo G, Pedrinelli R. Angiotensin II, tissue factor and the thrombotic paradox of hypertension. *Expert Rev Cardiovasc Ther*. 2010;8(12):1723–1729. doi:10.1586/erc.10.161 21108554

[44] Guo T, Fan Y, Chen M, et al. Cardiovascular implications of fatal outcomes of patients with coronavirus disease 2019 (COVID-19). *JAMA Cardiol*. 2020;5(7):811–818. doi:10.1001/jamacardio.2020.1017 32219356 PMC7101506

[45] Becker RC. COVID-19 update: covid-19-associated coagulopathy. *J Thromb Thrombolysis*. 2020;50(1):54-67. doi:10.1007/s11239-020-02134-3. PMC7225095 32415579

[46] Al-Ani F, Chehade S, Lazo-Langner A. Thrombosis risk associated with COVID-19 infection a scoping review. *Thromb Res*. 2020;192:152–160. doi:10.1016/j.thromres.2020.05.039 32485418 PMC7255332

[47] Zhang L, Feng X, Zhang D, et al. Deep vein thrombosis in hospitalized patients with COVID-19 in Wuhan, China: prevalence, risk factors, and outcome. *Circulation*. 2020;142(2):114–128. doi:10.1161/circulationaha.120.046702 32421381

[48] Yang J, Zheng Y, Gou X, et al. Prevalence of comorbidities and its effects in patients infected with SARS-CoV-2: a systematic review and meta-analysis. *Int J Infect Dis*. 2020;94:91–95. doi:10.1016/j.ijid.2020.03.017 32173574 PMC7194638

[49] Levi M, Thachil J, Iba T, Levy JH. Coagulation abnormalities and thrombosis in patients with COVID-19. *Lancet Haematol*. 2020;7(6):e438–e440. doi:10.1016/S2352-3026(20)30145-9 32407672 PMC7213964

[50] Thachil J, Tang N, Gando S, et al. ISTH interim guidance on recognition and management of coagulopathy in COVID-19. *J Thrombosis Haemostasis*. 2020;18(5):1023–1026. doi:10.1111/jth.14810 PMC9906133 32338827

[51] Nutescu EA, Burnett A, Fanikos J, Spinler S, Wittkowsky A. Pharmacology of anticoagulants used in the treatment of venous thromboembolism. *J Thromb Thrombolysis*. 2016;41(1):15–31. doi:10.1007/s11239-015-1314-3 26780737 PMC4715843

[52] Kim SY, Jin W, Sood A, et al. Characterization of heparin and severe acute respiratory syndrome-related coronavirus 2 (SARS-CoV-2) spike glycoprotein binding interactions. *Antiviral Res*. 2020;181:104873. doi:10.1016/j.antiviral.2020.104873 32653452 PMC7347485

[53] Thachil J. The versatile heparin in COVID-19. *J Thrombosis Haemostasis*. 2020;18(5):1020–1022. doi:10.1111/jth.14821 PMC9906146 32239799

[54] White D, MacDonald S, Bull T, et al. Heparin resistance in COVID-19 patients in the intensive care unit. *J Thromb Thrombolysis*. 2020;50(2):287–291. doi:10.1007/s11239-020-02145-0 32445064 PMC7242778

[55] Ortega‐Paz L, Capodanno D, Montalescot G, Angiolillo Dominick J. Coronavirus disease 2019–associated thrombosis and coagulopathy: review of the pathophysiological characteristics and implications for antithrombotic management. *J Am Heart Assoc*. 2021;10(3):e019650. doi:10.1161/JAHA.120.019650 33228447 PMC7955431

[56] Chen S, Zhang D, Zheng T, Yu Y, Jiang J. DVT incidence and risk factors in critically ill patients with COVID-19. *J Thromb Thrombolysis*. 2021;51(1):33–39. doi:10.1007/s11239-020-02181-w 32607652 PMC7324310

[57] Shah A, Donovan K, McHugh A, et al. Thrombotic and haemorrhagic complications in critically ill patients with COVID-19: a multicentre observational study. *Critical Care*. 2020;24(1):1–10. doi:10.1186/s13054-020-03260-3 32948243 PMC7499016

[58] Gibson CJ, Alqunaibit D, Smith KE, et al. Probative value of the d-dimer assay for diagnosis of deep venous thrombosis in the coronavirus disease 2019 syndrome. *Crit Care Med*. 2020;48(12):e1322–e1326. doi:10.1097/ccm.0000000000004614 32932347

[59] Monfardini L, Morassi M, Botti P, et al. Pulmonary thromboembolism in hospitalised COVID-19 patients at moderate to high risk by Wells score: a report from Lombardy, Italy. *Br J Radiol*. 2020;93(1113):20200407. doi:10.1259/bjr.20200407 32735448 PMC7465860

[60] Avruscio G, Camporese G, Campello E, et al. COVID‐19 and venous thromboembolism in intensive care or medical ward. *Clin Transl Sci*. 2020;13(6):1108–1114. doi:10.1111/cts.12907 32989908 PMC7567296

[61] Al-Samkari H, Karp Leaf RS, Dzik WH, et al. COVID-19 and coagulation: bleeding and thrombotic manifestations of SARS-CoV-2 infection. *Blood*. 2020;136(4):489–500. doi:10.1182/blood.2020006520 32492712 PMC7378457

[62] Demelo-Rodríguez P, Cervilla-Muñoz E, Ordieres-Ortega L, et al. Incidence of asymptomatic deep vein thrombosis in patients with COVID-19 pneumonia and elevated D-dimer levels. *Thromb Res*. 2020;192:23–26. doi:10.1016/j.thromres.2020.05.018 32405101 PMC7219400

[63] Piazza G, Campia U, Hurwitz S, et al. Registry of arterial and venous thromboembolic complications in patients with COVID-19. *J Am Coll Cardiol*. 2020;76(18):2060–2072. doi:10.1016/j.jacc.2020.08.070 33121712 PMC7588178

[64] Brosnahan SB, Jonkman AH, Kugler MC, Munger JS, Kaufman DA. COVID-19 and respiratory system disorders: current knowledge, future clinical and translational research questions. *Arterioscler Thromb Vasc Biol*. 2020;40(11):2586–2597. doi:10.1161/ATVBAHA.120.314515 32960072 PMC7571846

